# Young and Non-Smoking: An Unusual Case of Bronchial Carcinoid

**DOI:** 10.3390/diagnostics16142190

**Published:** 2026-07-14

**Authors:** Ancuța-Alina Constantin, Antonio-Andrei Cotea, Florin Dumitru Mihălțan

**Affiliations:** 1 Department of Cardio-Thoracic Pathology, “Carol Davila” University of Medicine and Pharmacy, 050474 Bucharest, Romania; ancuta-alina.constantin@umfcd.ro (A.-A.C.); florin.mihaltan@umfcd.ro (F.D.M.); 2 Institute of Pneumology “Marius Nasta”, 050159 Bucharest, Romania

**Keywords:** bronchial carcinoid, typical carcinoid, pulmonary neuroendocrine tumor, endobronchial lesion, airway obstruction, bronchoscopy, bilobectomy

## Abstract

We report the case of a 30-year-old female, non-smoker, with no known allergies or occupational exposures, presenting with a two-year history of recurrent stridor and wheezing, recently exacerbating over the past two months. Physical examination and routine laboratory tests were largely unremarkable. Pulmonary function tests were within normal limits, with no significant bronchodilator response. Chest CT revealed a 15 × 13 mm perihilar right-sided lesion adjacent to the right pulmonary artery. Bronchoscopy identified a nearly obstructive, highly vascularized endobronchial lesion at the origin of the right lower lobe bronchus, with involvement of the carina between the right middle and lower lobe bronchi. Histopathology confirmed a typical bronchial carcinoid. The patient underwent open right middle and lower lobe bilobectomy. What renders this case unusual is the combination of a prolonged two-year diagnostic delay with initial misdiagnosis as asthma, the critical anatomical location of the tumor at the bronchus intermedius with early involvement of the right middle lobe carina, and the consequent necessity of bilobectomy despite an otherwise low-risk, typical carcinoid histology. This case underscores the importance of considering endobronchial neoplasms in young, non-smoking patients with refractory or atypical airway symptoms, and highlights the role of integrated imaging and bronchoscopic evaluation in guiding individualized surgical planning.

**Figure 1:**
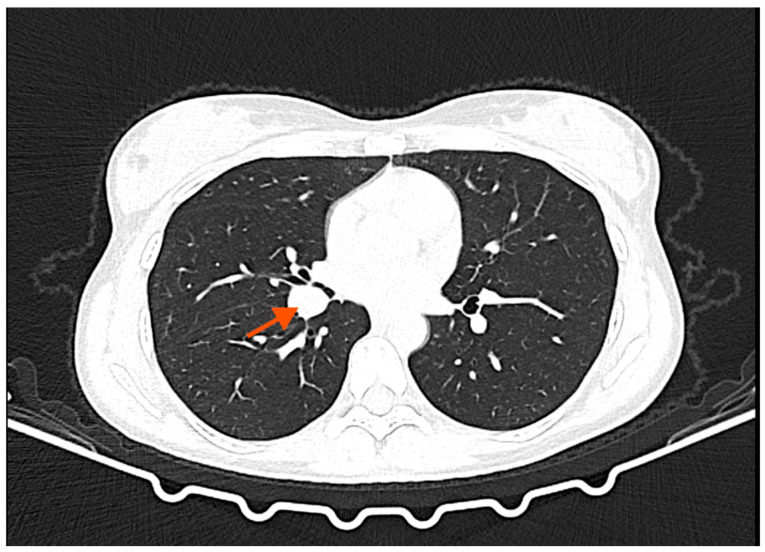
A 30-year-old female, non-smoker, with no known occupational exposures or significant comorbidities, was referred for pulmonology evaluation due to recurrent episodes of stridor and wheezing. Initially nocturnal, the symptoms progressively increased in frequency and began occurring during daytime, with notable exacerbation over the last two months. The patient denied fever, hemoptysis, weight loss, or other systemic symptoms. Notably, the symptoms had been present for approximately two years and had been attributed to asthma, for which the patient had received bronchodilator therapy without meaningful improvement—a common pattern of diagnostic delay in endobronchial neoplasms. Such a clinical presentation is consistent with the broad and often nonspecific respiratory manifestations described in bronchial carcinoid tumors, which may include wheezing, cough, dyspnea, or recurrent infections, although a subset of patients may remain asymptomatic [1]. Physical examination revealed a patient in good general condition, afebrile, with normal cardiovascular findings. Respiratory auscultation demonstrated expiratory wheezing, consistent with the symptomatic presentation, while no signs of respiratory distress or accessory muscle use were observed at rest. Routine laboratory investigations were within normal limits, except for a mild indirect hyperbilirubinemia of no clinical significance. Pulmonary function testing, including spirometry, plethysmography, and diffusion capacity, demonstrated values within predicted ranges, with no significant bronchodilator response (FVC 3.21 L [88%], FEV1 2.49 L [79%], FEV1/FVC 77.41%, PEF 4.31 L/s [61%], MEF-50 84.88%, DLCOc 82%, KCOc 94%). Notably, the peak expiratory flow was disproportionately reduced relative to FEV1 (PEF 61% vs. FEV1 79% predicted), a subtle spirometric asymmetry that, in retrospect, may reflect the influence of the central airway lesion on peak flow generation. The Empey index (FEV1[mL]/PEF[L/min]), calculated from these values, yielded 9.63—approaching but not exceeding the classical threshold of 10 for fixed upper airway obstruction, consistent with a lesion situated below the glottic level, where the index has reduced sensitivity compared to subglottic or tracheal pathology [2]. Despite the presence of wheezing and stridor, the absence of functional impairment suggested a localized obstructive process not detectable by standard pulmonary function testing, raising suspicion for a central airway lesion. Contrast-enhanced thoracic CT identified a well-circumscribed, moderately enhancing perihilar lesion in the right lung, measuring approximately 15 × 13 mm in the transaxial plane, situated adjacent to the division of the right pulmonary artery—anatomically corresponding to the level of the distal bronchus intermedius, just proximal to the origin of the right lower lobe bronchus. No mediastinal or hilar lymphadenopathy was observed, and no pleural or pericardial effusion was present. This pre-procedural CT characterization was of paramount importance in planning bronchoscopy, as it anticipated the critical relationship between the lesion and the right middle lobe carina. **The red arrow indicates the perihilar right-sided endobronchial lesion adjacent to the right pulmonary artery.** In this context, computed tomography plays a crucial role, as it typically demonstrates well-defined endobronchial or perihilar lesions with a round or ovoid configuration, sometimes associated with calcifications [3]. The imaging appearance raised the suspicion of an endobronchial tumor, with differential diagnoses including carcinoid tumor, bronchogenic carcinoma, or less likely benign lesions such as hamartoma.

**Figure 2:**
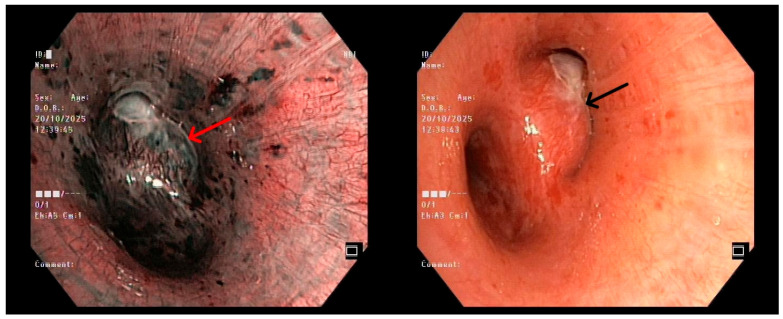
Flexible bronchoscopy was performed for further diagnostic clarification, providing direct visualization of the airway and enabling targeted tissue sampling. Of note, laryngeal examination at the outset of bronchoscopy revealed a tendency toward paradoxical, dyskinetic vocal cord motion—a finding that contributed to the patient’s stridor and prompted subsequent neurological evaluation, given the concurrently identified residual thymus (~4 cm) noted on thoracic surgical assessment. In this setting, bronchoscopy represents the key diagnostic modality, as it allows both direct visualization of the lesion and acquisition of tissue samples for histopathological confirmation [4]. Endoscopic examination revealed diffuse bronchitic changes throughout the trachea, left bronchial tree, right main bronchus, and right upper lobe bronchus. Moderate congestion was noted at the level of the distal bronchus intermedius. The right lower lobe bronchus was found to be subtotally obstructed at its origin and completely obstructed at the level of the basal trunk by a tumor mass with a broad base of implantation on the anterior wall of the bronchus intermedius at its most distal extent, with splaying of the carina between the right middle and lower lobe bronchi and partial engulfment of the right middle lobe orifice. The lesion exhibited well-defined smooth margins and a characteristic shiny, highly vascularized surface with hyperplastic vascular changes, features highly suggestive of bronchial carcinoid. Red and black arrows indicate the same endobronchial carcinoid lesion, visualized from two different endoscopic angles during the same procedure. Narrow Band Imaging (NBI) examination of the lesion surface demonstrated mucosal vascular abnormalities including hyperplastic neovascularization, further supporting the endoscopic impression of a well-differentiated endobronchial neoplasm. Furthermore, such lesions are characteristically described as smooth, polypoid, and highly vascularized, with an increased tendency to bleed during biopsy procedures [4]. In addition, the lesion appeared polypoid and friable, without evidence of ulceration, necrosis, or irregular infiltration of the surrounding mucosa. Therefore, while these findings favored a well-differentiated neuroendocrine tumor, more aggressive malignancies could not be completely excluded at this stage. Given the marked vascularity of the tumor and the associated risk of bleeding, bronchoscopic biopsy samples were obtained cautiously and in a limited number using conventional biopsy forceps, with careful procedural control. Moderate procedural bleeding occurred following biopsy, which was successfully controlled with bronchial toilet and local instillation of tranexamic acid (80 mg, four ampoules of 20 mL). Electrocautery or diathermy forceps were not available at our institution; these may offer a safer hemostatic alternative in highly vascular endobronchial lesions. The procedure was otherwise well tolerated, with no further immediate complications observed.

**Figure 3:**
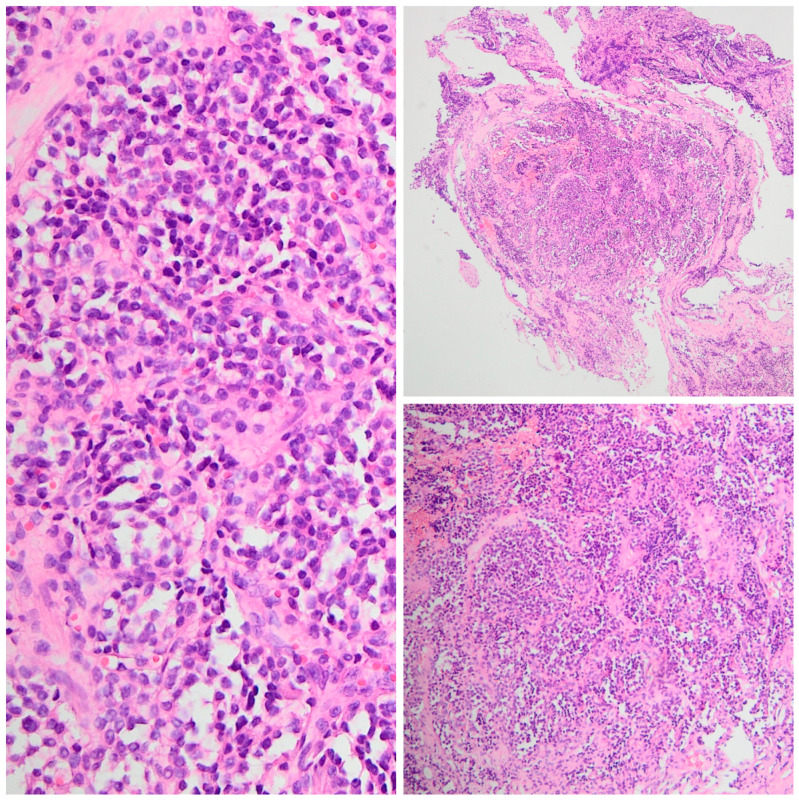
Microscopic examination of the bronchoscopic biopsy specimens revealed multiple fragments of bronchial mucosa infiltrated by a neoplastic proliferation composed of cells arranged predominantly in nests and trabecular patterns. The tumor cells were round to polygonal in shape, displaying relatively uniform, hyperchromatic nuclei with finely granular (“salt-and-pepper”) chromatin, a characteristic feature indicative of neuroendocrine differentiation. Furthermore, the cytoplasm was moderate in amount and poorly defined, contributing to the overall monotonous appearance of the tumor population. Importantly, no significant nuclear pleomorphism, necrosis, or increased mitotic activity was identified in the examined sections, findings that argue against a high-grade neuroendocrine carcinoma. In addition, the preserved architectural organization and lack of aggressive histological features further support the diagnosis of a well-differentiated neuroendocrine tumor, most consistent with a typical bronchial carcinoid. These histopathological findings are in accordance with established diagnostic criteria, according to which typical carcinoids are defined by fewer than 2 mitoses per 2 mm^2^ and absence of necrosis, whereas atypical carcinoids demonstrate higher mitotic activity and/or focal necrosis. Furthermore, typical carcinoids are characterized by a relatively low metastatic potential, with reported rates of lymph node involvement and distant metastasis remaining limited [5]. In addition, more recent classifications have incorporated supplementary phenotypic, molecular, and genetic features into the diagnostic framework of these tumors [6]. Moreover, complementary microbiological and cytological analyses of bronchial aspirates were negative, thereby excluding infectious processes as well as alternative malignant etiologies, and reinforcing the specificity of the histopathological diagnosis.

**Figure 4:**
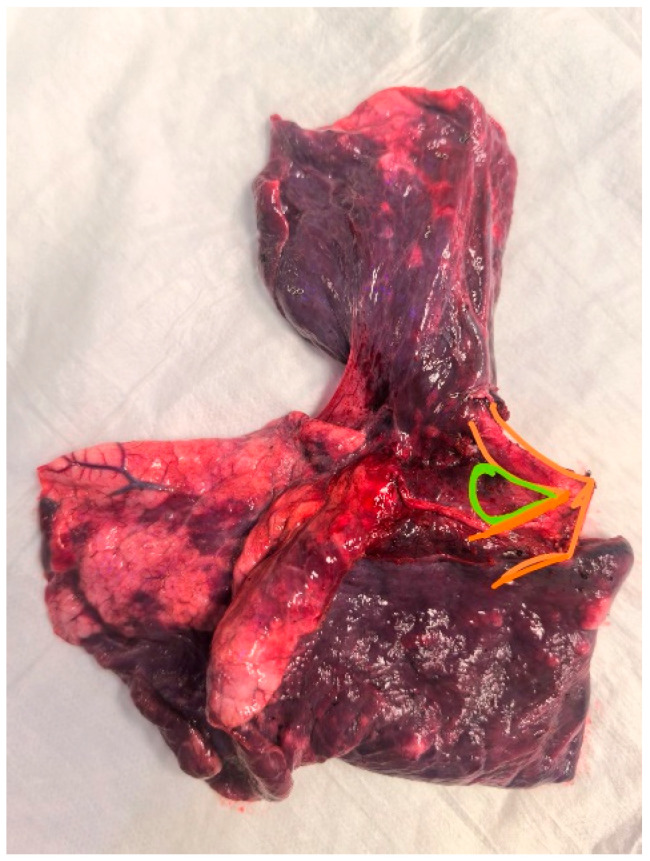
Prior to definitive surgical referral, the multidisciplinary team reviewed preoperative staging. Given the imaging and bronchoscopic appearance strongly consistent with a typical bronchial carcinoid—a low-grade tumor with limited metastatic potential—and the absence of mediastinal or hilar lymphadenopathy on CT, the risk of nodal or distant disease was considered low. Endobronchial ultrasound (EBUS) was not performed, as it was not available at the treating institution. Similarly, functional imaging with 68Ga-DOTATATE PET/CT or somatostatin receptor scintigraphy was not obtained, a decision informed by the absence of CT evidence of nodal involvement and the low pre-test probability of metastatic disease in this clinical profile. Given the central location of the tumor and its nearly obstructive nature, the patient was referred for surgical management. In this context, surgical resection is considered the treatment of choice for localized bronchial carcinoid tumors [7]. Although parenchyma-sparing techniques—such as sleeve resection or bronchotomy—are preferred whenever anatomically feasible, the extent of this tumor precluded such an approach: the broad-based implantation at the anterior wall of the distal bronchus intermedius with involvement of the right middle lobe carina and partial engulfment of the right middle lobe orifice meant that a margin-negative, parenchyma-sparing resection could not be safely achieved. Thus, in accordance with current recommendations for localized bronchial carcinoid tumors and after thoracic surgical evaluation, a right middle and lower lobe bilobectomy (RML + RLL) was performed via open thoracotomy. The choice of surgical technique is primarily determined by tumor location and extent, with options ranging from parenchyma-sparing procedures to more extensive resections such as lobectomy or bilobectomy [8]. Intraoperatively, the findings confirmed the presence of a well-localized endobronchial mass, without evidence of pleural dissemination, adjacent parenchymal invasion, or mediastinal involvement, thereby supporting the feasibility of a curative approach. Furthermore, systematic exploration and lymph node assessment did not reveal any suspicious metastatic disease, and complete surgical resection was successfully achieved. Subsequently, gross examination of the resected specimen confirmed a well-circumscribed intrabronchial tumor with a firm consistency and homogeneous cut surface, without areas of hemorrhage or necrosis. In addition, the final histopathological analysis of the surgical specimen confirmed the diagnosis of a typical carcinoid tumor, demonstrating well-differentiated neuroendocrine morphology, clear resection margins, and absence of lymph node involvement or aggressive histological features, thereby indicating a favorable prognosis. Following complete resection, adjuvant therapy is generally not indicated in cases of typical carcinoid tumors [9]. Consistent with these findings, reported survival outcomes are favorable, with 5-year survival rates exceeding 90% for typical bronchial carcinoids [8].

**Figure 5:**
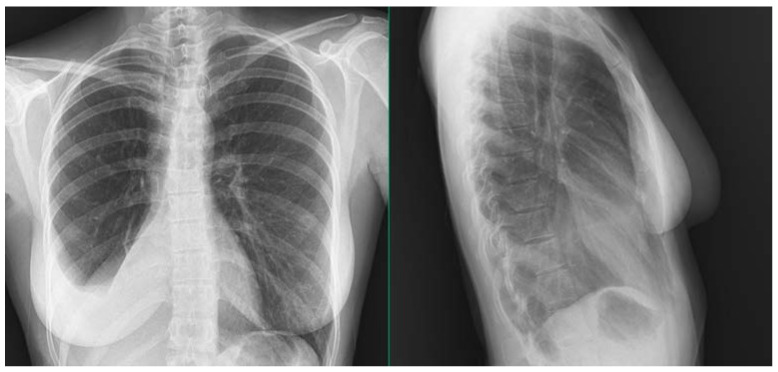
Approximately two months after surgery, the patient was readmitted due to persistent respiratory symptoms, including a productive cough with seromucous sputum, exertional dyspnea, and dysphonia. However, she remained afebrile and in good general condition, with no signs of acute systemic infection. Chest radiography performed at this stage demonstrated postoperative changes consistent with a right middle and lower lobe bilobectomy. In addition, a right basal opacity with fluid characteristics was identified, suggestive of a small pleural effusion, while further findings included signs of compensatory pulmonary hyperinflation in the remaining lung parenchyma. Moreover, pulmonary function tests revealed a mild distal obstructive ventilatory defect, accompanied by a slight reduction in diffusing capacity for carbon monoxide (FVC 3.02 L [83%], FEV1 2.30 L [73%], FEV1/FVC 76.29%, PEF 4.62 L/s [65%], MEF-50 47%, DLCOc 69%, KCOc 86%), representing a meaningful functional decline compared to the pre-operative baseline (FEV1 79%, DLCO 82% predicted). These functional impairments are most likely attributable to postoperative anatomical alterations and adaptive physiological changes following lung resection, rather than indicating tumor recurrence or significant disease progression.

**Figure 6:**
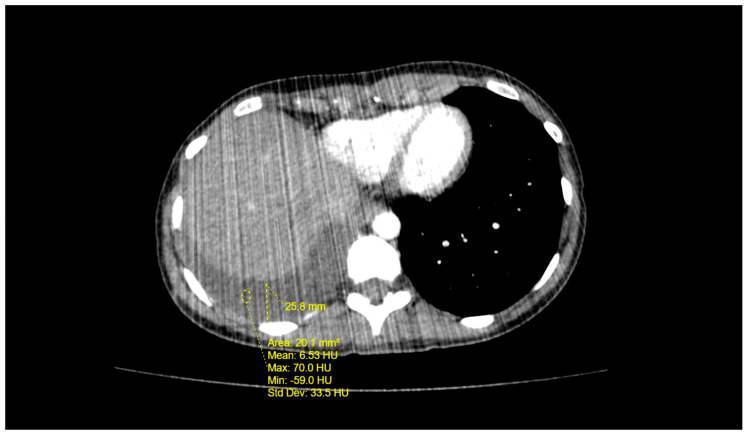
Thoracic CT examination confirmed the presence of a small right-sided pleural effusion, measuring approximately 25 mm in thickness. However, there was no evidence of bronchial stump dehiscence, local tumor recurrence, or mediastinal pathology, thereby supporting a favorable postoperative status. In addition, a ground-glass nodular opacity, measuring approximately 15 × 12 mm, was identified in the posterior segment of the left upper lobe, a finding that warranted careful radiological follow-up to exclude potential neoplastic or inflammatory etiology. Furthermore, follow-up bronchoscopy demonstrated a short, well-healed, and intact bronchial stump, with no endoscopic signs of local recurrence. Importantly, NBI examination of the bronchial mucosa at the level of the stump and surrounding airways showed no vascular abnormalities or mucosal changes, providing additional endoscopic confirmation of the absence of local tumor recurrence at two months post-resection. Diffuse bronchitic changes persisted, accompanied by minimal endobronchial secretions, consistent with ongoing airway inflammation. Microbiological analysis of bronchial aspirate subsequently identified methicillin-sensitive Staphylococcus aureus (MSSA), in the context of pre-existing minimal bilateral cylindrical bronchiectases, and targeted antibiotic therapy was initiated, resulting in a favorable clinical and symptomatic evolution. Finally, the pleural effusion was interpreted as postoperative in origin, given its small volume and the absence of associated inflammatory or compressive features, no invasive intervention was required, and a conservative management approach was adopted. Overall, these findings highlight the favorable postoperative course in a case of typical bronchial carcinoid. They also underscore the importance of a systematic, multidisciplinary approach integrating clinical, radiologic, bronchoscopic, and histopathological data for accurate diagnosis, appropriate surgical management, and optimal patient outcomes, while emphasizing the need for continued follow-up to monitor for potential recurrence or associated pulmonary abnormalities.

## Data Availability

The original contributions presented in this study are included in the article. Further inquiries can be directed to the corresponding author.
